# Impact of Mephedrone on Fear Memory in Adolescent Rats: Involvement of Matrix Metalloproteinase-9 (MMP-9) and N-Methyl-D-aspartate (NMDA) Receptor

**DOI:** 10.3390/ijms24031941

**Published:** 2023-01-18

**Authors:** Pawel Grochecki, Irena Smaga, Karolina Wydra, Marta Marszalek-Grabska, Tymoteusz Slowik, Ewa Kedzierska, Joanna Listos, Ewa Gibula-Tarlowska, Malgorzata Filip, Jolanta H. Kotlinska

**Affiliations:** 1 Department of Pharmacology and Pharmacodynamics, Medical University, Chodzki 4A, 20-093 Lublin, Poland; 2 Department of Drug Addiction Pharmacology, Maj Institute of Pharmacology, Polish Academy of Sciences, Smetna 12, 31-343 Krakow, Poland; 3 Department of Experimental and Clinical Pharmacology, Medical University, Jaczewskiego 8B, 20-090 Lublin, Poland; 4 Experimental Medicine Center, Medical University, Jaczewskiego 8, 20-090 Lublin, Poland

**Keywords:** mephedrone, PTSD, fear conditioning, conditioned place preference, adolescence male rats

## Abstract

Treatment of Post-Traumatic Stress Disorder (PTSD) is complicated by the presence of drug use disorder comorbidity. Here, we examine whether conditioned fear (PTSD model) modifies the rewarding effect of mephedrone and if repeated mephedrone injections have impact on trauma-related behaviors (fear sensitization, extinction, and recall of the fear reaction). We also analyzed whether these trauma-induced changes were associated with exacerbation in metalloproteinase-9 (MMP-9) and the GluN2A and GluN2B subunits of N-methyl-D-aspartate (NMDA) glutamate receptor expression in such brain structures as the hippocampus and basolateral amygdala. Male adolescent rats underwent trauma exposure (1.5 mA footshock), followed 7 days later by a conditioned place preference training with mephedrone. Next, the post-conditioning test was performed. Fear sensitization, conditioned fear, anxiety-like behavior, extinction acquisition and relapse were then assessed to evaluate behavioral changes. MMP-9, GluN2A and GluN2B were subsequently measured. Trauma-exposed rats subjected to mephedrone treatment acquired a strong place preference and exhibited impairment in fear extinction and reinstatement. Mephedrone had no effect on trauma-induced MMP-9 level in the basolateral amygdala, but decreased it in the hippocampus. GluN2B expression was decreased in the hippocampus, but increased in the basolateral amygdala of mephedrone-treated stressed rats. These data suggest that the modification of the hippocampus and basolateral amygdala due to mephedrone use can induce fear memory impairment and drug seeking behavior in adolescent male rats.

## 1. Introduction

Post-Traumatic Stress Disorder (PTSD) is psychiatric illness triggered by shocking, dangerous, or life-threatening events, that are either experienced or witnessed [1]. Characteristic symptoms of PTSD are the appearance of, among others, anxiety, and a sense of threat in response to a previously acquired stimulus. Recently, the understanding of the pathophysiology of PTSD has been advanced by including the analysis of learning mechanisms related to fear conditioning and extinction [2,3]. According to conditioning theories, PTSD symptoms are caused by fear responses, such as avoidance, that occur when exposed to trauma-related cues. Additionally, maintenance of PTSD symptoms is hypothesized to involve disruption of fear extinction processes [4].

High rates of comorbidities suggest that PTSD and substance use disorders (alcohol, nicotine, cocaine) are functionally related to one another [5,6,7], and such comorbidities can alter the development, maintenance and treatment of these disorders [7]. Furthermore, recently, it has been reported that adolescents with a history of traumatic events have found to be self-dosing with 3,4-methylenedioxymethamphetamine (MDMA) after the first occurrence of PTSD symptoms [8]. Of note, many recent papers show that MDMA could be useful in PTSD treatment [9]. Stress can modulate the initial rewarding effects of additive drugs, reinstate drug seeking, and cause relapse to substance use. On the other hand, substance use can alter biological response to stress [10,11,12], thus changing stress responses in addicted individuals. These drug effects are further complicated by the many demonstrations that abuse substances have effects on memory. These effects can include promoting or impairing memory, depending on the receptor systems, and signaling cascades that the substance affects [7].

Mephedrone (4-methylmethcathinone) is a semi-synthetic derivative of cathinone, a psychoactive compound found in the leaves of *Catha edulis*. Cathinone derivatives show great structural similarity to neurotransmitters such as adrenaline, noradrenaline and dopamine, as well as exogenous substances classified as psychostimulants: amphetamine, methamphetamine and MDMA (ecstasy). The pharmacological similarity of mephedrone to the illegal MDMA was one of the most important reasons for the widespread popularity of mephedrone among young users, especially those participating in long-hour club events. Mephedrone is abused by club drug users, predominantly adolescents and young adults [13,14], in a binge-like fashion [15] to induced reward effects. Long-term mephedrone consumption drives addiction potential and dependency symptoms, cravings and withdrawal syndromes [16]. Our previous preclinical studies confirm that mephedrone possesses abuse potential and shows deleterious impact on memory processes. In these effects of mephedrone, both the matrix metalloproteinase-9 (MMP-9) and N-methyl-D-aspartate (NMDA) receptors are engaged [17,18]. The negative effect of mephedrone on memory processes is particularly strong when mephedrone is combined with ethanol [18,19].

MMP-9 is a proteolytic enzyme zinc-dependent endopeptidase that is expressed throughout the body, mediating both physiological (e.g., tissue growth) and pathological (e.g., stroke) processes. In the central nervous system (CNS), MMP-9 is responsible for the remodeling of nervous tissue, which enables neurogenesis, dendritic tree expansion, synaptogenesis and myelination. MMP-9 is one of the key enzymes involved in the processes of neuroplasticity, therefore, its participation is essential in learning and memory processes, and changes in its expression have also been described in states associated with the experience of severe stress, which then triggered pathological reactions within the CNS [20]. MMP-9 has emerged as a physiological regulator of NMDA receptor dependent synaptic plasticity and memory [21].

Glutamatergic transmission is engaged in the pathophysiology of PTSD, particularly in the effects on NMDA signaling in the synaptic plasticity underlying learning and memory [22]. NMDA receptors are comprised of two GluN1 subunits and two GluN2 (A–D) and GluN3 (A, B) subunits. In adults, GluN2A and GluN2B are the main subunit receptor complexes with GluN1 at excitatory synapses. GluN2B-containing NMDA receptors play a preferential role in inducing synaptic plasticity, which is critical for the extinction of fear memories [23]. Systemic injection of GluN2B-specific NMDA antagonists ((RS)-3-(2-carboxypiperazin-4-yl)-propyl-1-phosphonic acid, ifenprodil) can impair the retention of fear extinction learning. GluN2B-containing NMDA receptors in both the amygdala and medial prefrontal cortex are also involved in reducing fear during extinction, whereas GluN2A-containing NMDA receptors play a greater role in the initial formation and/or stabilization of learned fear [23]. Rodent studies demonstrate that GluN2B subunit-containing NMDA receptors play pivotal roles in fear extinction learning.

Fear conditioning is one of the leading animal models used in PTSD research [24,25,26]. This task measures both hippocampus-dependent and hippocampus-independent learning [27,28,29,30]. Learning to associate the context with the shock unconditional stimulus (US) is hippocampus-dependent and amygdala-dependent [28,30]. For the hippocampus-dependent part of the task, animals (rats, mice) are required to use stimuli from the environment to form the association with the shock. In contrast, the hippocampus-independent portion of the task requires an association to be formed between an auditory conditioned stimulus (CS) and the shock. This is dependent on amygdala function [28].

**Figure 1:**
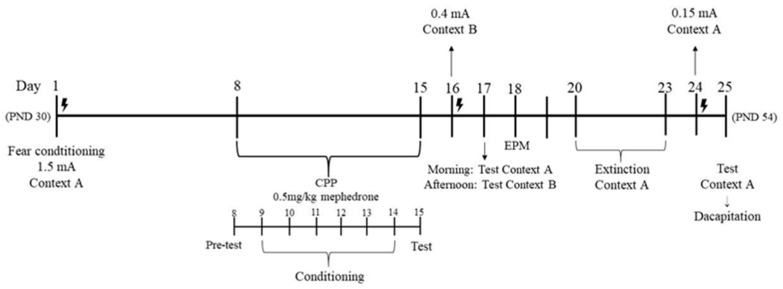
Experimental design.

The aim of the current study was to evaluate bidirectionality of trauma and reward system functioning in adolescent rats. For this purpose, adolescent rats (PND30) were exposed to high intensity (1.5 mA) footshock in a fear conditioning chamber (PTSD model [25,26]). Next, the animals were subjected to the mephedrone-induced conditioned place preference (CPP). We assessed: (1) whether conditioned fear (PTSD model) modifies the rewarding effect of mephedrone, and (2) if repeated mephedrone injections have impact on trauma-related behaviors (fear sensitization, extinction, and recall of the fear reaction), or (3) whether NMDA receptor subunits (GluN1, GluN2B) and MMP-9 in such brain structures as the hippocampus (HIPP) and basolateral amygdala (BLA) are involved in these behavioral effects (Figure 1).

## 2. Results

### 2.1. Stage 1: Impact of Footshock Stress on the Acquisition of Mephedrone-Induced CPP in Adolescent Rats

Two-way ANOVA of the results presented as the difference between the time of animals spent in the compartment associated with the mephedrone administration on the day of the Test and the Pre-test (score) showed the statistically significant effect of the mephedrone factor [F (1,24) = 3.476, *p* < 0.0001] and stress factor [F (1,24) = 7.880, *p* < 0.01], without the statistically significant interactions between them [F (1,24) = 3.476, *p* > 0.05].

**Figure 2:**
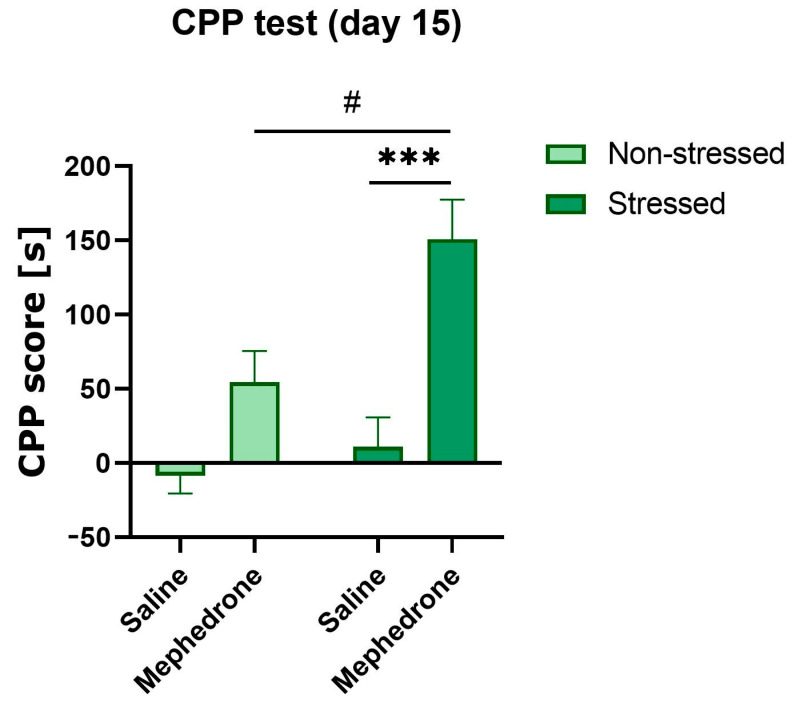
Impact of fear conditioning (PTSD-like symptoms) on the acquisition of mephedrone (5 mg/kg)—induced CPP in adolescent rats. Data are expressed as scores (the differences between post-test and test time spent in the drug-associated compartment). Data represent mean ± SEM (*N* = 7 rats/group). ^#^ *p* < 0.05 Stressed/Mephedrone vs. Stressed/Saline; *** *p* < 0.001 Stressed/Mephedrone vs. Non-stressed/Mephedrone.

The Bonferroni’s multiple comparisons test showed statistically significant differences in preference score between previously stressed animals receiving mephedrone during conditioning, and stressed animals receiving saline (*p* < 0.001), and between stressed animals receiving mephedrone and animals that also received mephedrone, but were not previously stressed (*p* < 0.05) (Figure 2).

### 2.2. Stage 2: Impact of Repeated Mephedrone Injections on Fear Sensitization in Adolescent Rats. Anxiety-like Behavior Based on an Elevated Plus-Maze Test

**Figure 3:**
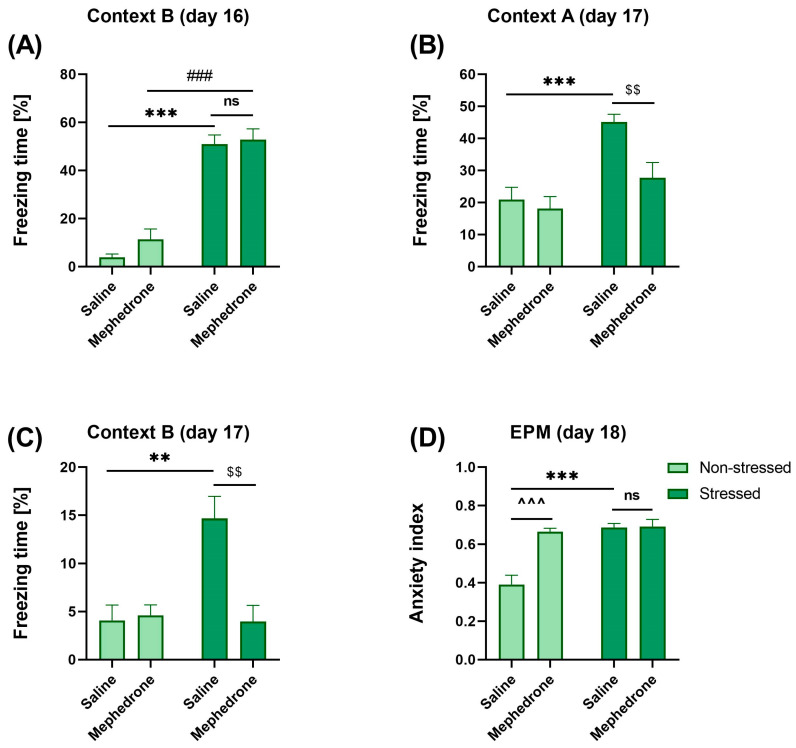
Impact of repeated mephedrone (5 mg/kg) injections on fear conditioning (PTSD-like syndrome) in adolescent rats: (**A**) sensitization of freezing behavior in context B (day 16); (**B**) freezing behavior in context A (day 17, morning); (**C**) freezing behavior in context B (day 17, afternoon) and (**D**) anxiety index recorded during the EPM test. Data represent mean ± SEM (*N* = 7 rats/group). ** *p* < 0.01 Stressed/Saline vs. Non-stressed/Saline; *** *p* < 0.001 Stressed/Saline vs. Non-stressed/Saline; ^###^ *p* < 0.001 Stressed/Mephedrone vs. Non-stressed/Mephedrone; ^$$^ *p* < 0.01 Stressed/Saline vs. Stressed/Mephedrone; ^^^^^ *p* < 0.001 Non-stressed/Saline vs. Non-stressed/Mephedrone.

Two-way ANOVA of the freezing time of the animals in context B (day 16, 0.4 mA footshock) revealed the statistically significant influence of the stress factor [F (1,24) = 141.4, *p* < 0.001]. Two-way ANOVA, however, did not show the significant effect of mephedrone administration [F (1,24) = 1.577, *p* > 0.05], nor interaction between these factors [F (1,24) = 0.5563, *p* > 0.05]. The Bonferroni post-hoc test showed statistically significant differences in the freezing time between the stressed animals that received mephedrone in the CPP procedure and non-stressed animals that also received mephedrone (*p* < 0.001). Furthermore, significant differences were found between stressed animals receiving saline in the CPP procedure and control animals that received saline, but were not stressed (*p* < 0.001). Differences between mephedrone-treated and saline-treated stressed animals were not significant (Figure 3A). Two-way ANOVA of the freezing time of the animals in the context of A (day 17, no footshock) showed the statistically significant effect of the stress [F (1,24) = 19.72, *p* < 0.001] and the mephedrone factor [F (1,24) = 7.100, *p* < 0.05]. Two-way ANOVA, however, did not show statistically significant interaction between these factors [F (1,24) = 3.721, *p* > 0.05]. The Bonferroni’s multiple comparisons test showed statistically significant differences in the freezing time between stressed animals that received mephedrone in the CPP procedure and stressed animals that received saline (*p* < 0.01). Significant differences were also observed between stressed animals that received saline and rats that received vehicle but were not previously stressed (*p* < 0.001) (Figure 3B).

Two-way ANOVA of the freezing time of the animals in the context of B (day 17, no footshock) showed the statistically significant effect of the stress [F (1,24) = 8.327, *p* < 0.001], mephedrone factor [F (1,24) = 8.714, *p* < 0.01] and interaction between these factors F (1,24) = 10.67, *p* < 0.01]. The Bonferroni’s multiple comparisons test showed statistically significant differences in the freezing time between stressed animals that received mephedrone in the CPP procedure and stressed animals that received saline (*p* < 0.01). Significant differences were also observed between stressed animals that received saline and rats that received vehicle, but were not previously stressed (*p* < 0.01) (Figure 3C).

Two-way ANOVA of the results obtained in the EPM (day 18) calculated as the anxiety index indicated a statistically significant influence of the stress [F (1,24) = 23.78, *p* < 0.001], mephedrone factor [F (1,24) = 17.94, *p* < 0.001] and significant interaction between these factors [F (1,24) = 16.60, *p* < 0.001]. The Bonferroni’s multiple comparisons test showed statistically significant differences in the anxiety index between non-stressed animals that received mephedrone and non-stressed animals that received saline (*p* > 0.001). Post-hoc test also revealed a significant difference between stressed animals that received saline and the animals that also received saline but were not previously stressed (*p* < 0.001). Differences between mephedrone-treated and saline-treated stressed animals were not significant (Figure 3D).

### 2.3. Stage 3: Impact of Repeated Mephedrone Injections on Extinction, Recall, and Retention of Fear Memory

**Figure 4:**
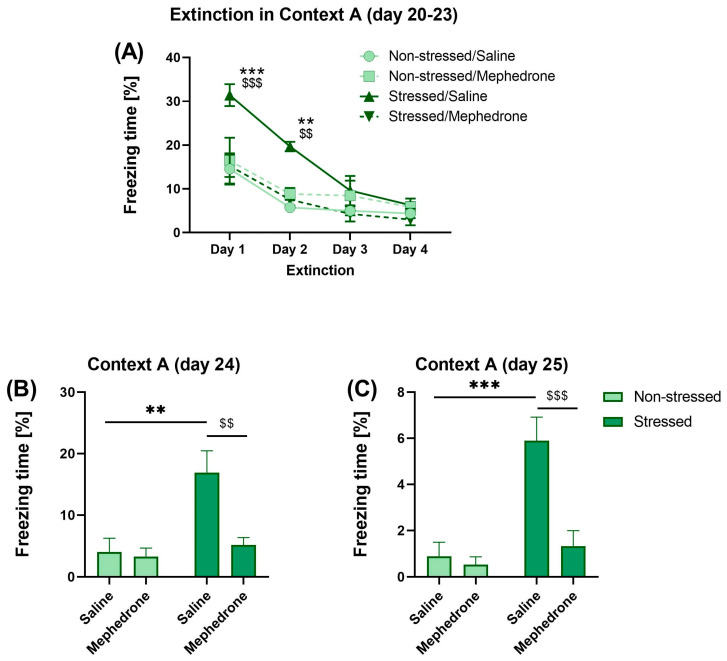
Impact of repeated mephedrone (5 mg/kg) injections on: (**A**) extinction; (**B**) recall (day 24) and (**C**) retention (day 25) of fear memory in PTSD-like model in adolescent rats. Data represent mean ± SEM (*N* = 7 rats/group). ** *p* < 0.01 Non-stressed/Saline vs. Stressed/saline; *** *p* < 0.001 Non-stressed/Saline vs. Stressed/Saline; ^$$^ *p* < 0.01 Stressed/Saline vs. Stressed/Mephedrone; ^$$$^ *p* < 0.001 Stressed/Saline vs. Stressed/Mephedrone.

Three-way ANOVA of the results obtained in the extinctions phase (Days 20–23) indicated a statistically significant effect of the stress [F (1,96) = 8.409, *p* < 0.01], mephedrone [F (3,96) = 7.888, *p* < 0.01], time factor [F (3,96) = 28.94, *p* < 0.01], stress x time [F (3,96) = 3086, *p* < 0.05] and stress x mephedrone [F (1,96) = 23.86, *p* < 0.001] interactions. The Bonferroni’s multiple comparisons test showed statistically significant differences in the freezing time measured on day 20 (*p* < 0.001; 1st day of extinction) and day 21 (*p* < 0.01; 2nd day of extinction) of the experiment between the stressed animals that received mephedrone in the CPP procedure and non-stressed animals that received saline. Statistically significant differences were also observed on day 20 (*p* < 0.001; 1st day of extinction) and day 21 (*p* < 0.01; 2nd day of extinction) of the experiment between the stressed animals that received saline in the CPP procedure and non-stressed animals that also received saline (Figure 4A).

Two-way ANOVA of the freezing time of the animals in context A (day 24, 0.15 mA foot-shock) showed the statistically significant influence of the stress factor [F (1,24) = 10.42, *p* < 0.01], the mephedrone factor [F (1,24) = 7.45, *p* < 0.05] and interactions between them [F (1,24) = 5.805, *p* < 0.05]. The Bonferroni’s multiple comparisons test showed statistically significant differences in the time of freezing between the stressed animals that received mephedrone in the CPP procedure, and stressed animals that received saline (*p* < 0.01). Post-hoc test also revealed significant difference between stressed animals that received saline in the CPP procedure and animals that also received saline, but were not previously stressed (*p* < 0.01) (Figure 4B).

Two-way ANOVA of the freezing time of the animals in the context of A (day 25, no foot-shock) showed the statistically significant influence of the stress factor [F (1,24) = 17.17, *p* < 0.001], mephedrone factor [F (1,24) = 12.39, *p* < 0.01] and interaction between these factors [F (1,24) = 8.942, *p* < 0.01]. The Bonferroni’s multiple comparisons test showed statistically significant differences in the freezing time between the stressed animals that received mephedrone in the CPP procedure and stressed animals that received saline (*p* < 0.001). Post-hoc test also revealed significant difference between stressed animals that received saline in the CPP procedure and animals that also received a saline, but were not previously stressed (*p* < 0.001) (Figure 4C).

### 2.4. ELISA Assays: Impact of Footshock Stress and Repeated Mephedrone Injections on the Expression of NMDA Receptor Subunits (GluN2A and GluN2B) and MMP-9 in the HIPP and BLA

**Figure 5:**
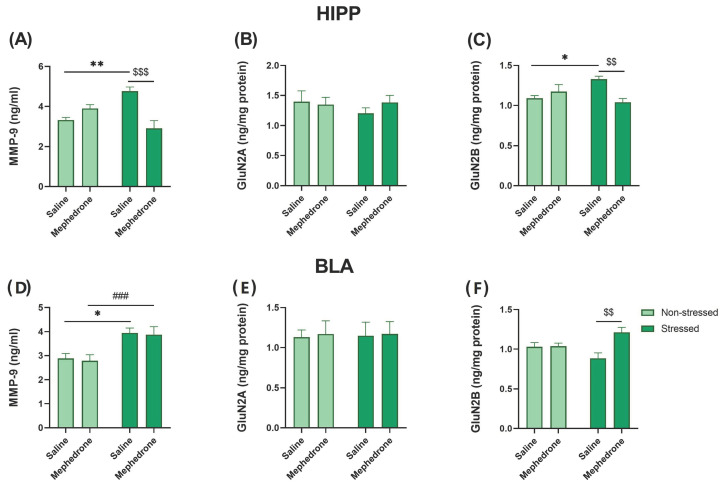
Impact of repeated mephedrone (5 mg/kg) injections on: the expression of (**A**) MMP-9; (**B**) GluN2A and (**C**) GluN2B in the HIPP, as well as (**D**) MMP-9; (**E**) GluN2A and (**F**) GluN2B in the BLA in the fear conditioned rats. Data represent mean ± SEM (*N* = 7 rats/group). * *p* < 0.05 Non-stressed/Saline vs. Stressed/saline; ** *p* < 0.01 Non-stressed/Saline vs. Stressed/saline; ^###^ *p* < 0.001 Stressed/Mephedrone vs. Non-stressed/Mephedrone; ^$$^ *p* < 0.01 Stressed/Saline vs. Stressed/Mephedrone; ^$$$^ *p* < 0.001 Stressed/Saline vs. Stressed/Mephedrone.

Two-way ANOVA show significant effect of repeated mephedrone administration [F (1,24) = 6.430, *p* < 0.05], but not footshock stress [F (1,24) = 0.7969, *p* > 0.05] on the MMP-9 expression in the HIPP. However, two-way ANOVA revealed significant interaction of these factors [F (1,24) = 23.49, *p* < 0.001]. In addition, Bonferroni’s multiple comparisons test showed statistically significant differences in the hippocampal MMP-9 expression between stressed animals that received mephedrone and stressed animals that received saline (*p* < 0.001). Post-hoc test also revealed significant differences between stressed and non-stressed saline-treated animals (*p* < 0.01) (Figure 5A).

Furthermore, two-way ANOVA did not show any significant effect of repeated mephedrone administration [F (1,24) = 0.2494, *p* > 0.05], footshock [F (1,24) = 0.3456, *p* > 0.05] nor interactions between these factors [F (1,24) = 0.7664, *p* > 0.05] on GluN2A expression in the HIPP. Moreover, Bonferroni’s multiple comparisons test did not reveal any significant differences between groups (Figure 5B).

Moreover, two-way ANOVA revealed no significant effect of footshock stress [F (1,24) = 0.96, *p* > 0.05], nor repeated mephedrone administration [F (1,24) = 3.418, *p* > 0.05] on GluN2B expression in the HIPP. However, two-way ANOVA showed significant interaction of these factors [F (1,24) = 11.22, *p* < 0.01]. Bonferroni’s multiple comparisons test revealed significant differences in the hippocampal GluN2B expression between the stressed saline- and mephedrone-treated (*p* < 0.01) rats, and saline-treated non-stressed and stressed (*p* < 0.05) rats (Figure 5C).

Two-way ANOVA did not show any significant effect of repeated mephedrone administration [F (1,24) = 10.96, *p* > 0.05] on the MMP-9 expression in the BLA. However, two-way ANOVA revealed significant impact of footshock [F (1,24) = 18.31, *p* < 0.001], but no interaction between these factors [F (1,24) = 0.0035, *p* > 0.05]. In addition, Bonferroni’s multiple comparisons test showed statistically significant differences in the MMP-9 expression in the BLA between non-stressed and stressed saline-treated animals. Furthermore, post-hoc test revealed significant differences between non-stressed and stressed mephedrone-treated rats (Figure 5D).

What is more, two-way ANOVA did not show any significant effect of repeated mephedrone administration [F (1,24) = 0.044, *p* > 0.05], footshock [F (1,24) = 0.0043, *p* > 0.05] nor interactions between these factors [F (1,24) = 0.0028, *p* > 0.05] on GluN2A expression in the BLA. Also, Bonferroni’s multiple comparisons test did not reveal any significant differences between groups (Figure 5E).

Finally, two-way ANOVA revealed significant effect of repeated mephedrone administration [F (1,24) = 8.862, *p* < 0.01], but no significant effect of footshock [F (1,24) = 0.0741, *p* > 0.05] on GluN2B expression in the BLA. Moreover, two-way ANOVA showed significant interaction of these factors [F (1,24) = 8.106, *p* > 0.01]. Bonferroni’s multiple comparisons test revealed significant differences in the GluN2B expression in the BLA between stressed saline- and mephedrone-treated rats (*p* < 0.01) (Figure 5F).

## 3. Discussion

The present study provides evidence for fear conditioning extinction in trauma-exposed (footshock stressed) adolescent rats subjected to repeated mephedrone administrations. The results suggest that repeated mephedrone administration produced impairment of fear conditioning memory. Moreover, trauma-exposed rats showed stronger mephedrone-induced CPP, indicating that these rats were more vulnerable to addictions. We found that the repeated mephedrone administration induces changes in MMP-9 and the GluN2B subunit of the NMDA receptor in the HIPP and BLA that could have impact on rat behavior.

### 3.1. Acquisition of Mephedrone-Induced CPP in Rats with Footshock Stress

Adverse life experiences may render individuals more prone to abuse addictive substances [31,32,33,34]. In experimental animals, it has been demonstrated that exposure to stressors (i.e., social defeat stress, social isolation, maternal separation, immobilization stress, footshocks stress, etc.) can produce behavioral and neurochemical adaptations that render animals more vulnerable to initiation, maintenance and escalation of drug consumption [34,35,36,37,38]. These effects were observed either with the CPP paradigm [39], with the two-bottle free choice paradigm [40,41] or with the self-administration paradigm [42]. However, other authors showed that stress does not necessarily lead to potentiate the effect of the drug or increase its consumption, for such drugs as cocaine [43,44] or methamphetamine [45]. The findings indicate that stress-induced drug consumption seems to be dependent on the type of stress applied [46,47]. Our data indicated striking association between footshock trauma and mephedrone-induced CPP procedure. The trauma-exposed animals subjected to the mephedrone CPP procedure developed more significant preference for the mephedrone-paired compartment than non-stressed rats. Our results are in accordance with the outcomes of previous studies demonstrating that stress enhances amphetamine and cocaine CPP [48,49,50] and intake of these drugs [51,52]. Furthermore, prior exposure to stress seems to increase the rewarding effects of MDMA in the CPP paradigm in adolescent mice [53]. Thus, our data suggest that trauma exposed adolescent rats are more vulnerable to mephedrone addiction.

### 3.2. Impact of Repeated Mephedrone Exposure on Fear Sensitization and Anxiety-like Behavior

Fear sensitization is a form of non-associate learning and a hallmark of PTSD [54]. It can be assessed in rodents (mice, rats) by measuring acoustic startle response [55], by exposing them to a neutral tone in a neutral chamber [54] or by conditioning animals using a low intensity footshock, and re-exposing them, at least 24 h later, to the conditioning context [56]. So far, published data showed that the stressed group displays more fear (fear sensitization) compared with the non-stressed group [57,58,59]. Our results confirm the latest data. The repeated saline or mephedrone (5 mg/kg) administration did not have an impact on freezing behavior in non-stressed animals. However, stressed animals show sensitization to lower (0.4 mA) aversive stimulus in a new context (context B). This sensitization is non-associative because it does not depend on a CS and US association. These results suggest that prior exposure to a shock stress, independent of a contextual fear memory, enhances subsequent fear memory when footshock is present. Furthermore, the repeated mephedrone administrations did not potentiate this fear response when the shock is present, but decreases freezing behavior on the next exposure (next day), suggesting a deleterious effect of mephedrone on the conditioning memory. Additionally, footshock stressed animals that were mephedrone- but not saline-treated and re-exposed to a trauma context without footshock showed a significant decrease in freezing time (Figure 3B). These data suggest that repeated mephedrone administration produces retrograde amnesia in trauma-exposed animals.

Like fear sensitization, anxiety is often reported in PTSD patients [60]. Interestingly, published results indicated that anxiety sensitivity and PTSD symptom severity were reciprocally related such that anxiety sensitivity predicted subsequent PTSD symptom severity and symptom severity predicted later anxiety sensitivity. Such findings have both theoretical and clinical implications [61]. Our EPM results indicated that stressed animals exhibit increased anxiety compared to non-stressed rats. Pre-exposure to mephedrone did not modify anxiety index in stressed animals, but it increased this index in non-stressed rats. These results suggest that mephedrone possesses anxiogenic-like properties. Published data demonstrated that synthetic cathinones, in addition to their addictive potential, often cause adverse psychiatric sequelae, including anxiety [62,63]. However, repeated psychostimulant administration induces anxiety-related behavior after 1 to 8 days of withdrawal [64,65,66]. Yet, another study demonstrated that chronic administration of psychostimulants such as methamphetamine or 4-metylethcathinone can reduce anxiety in EPM after two weeks of withdrawal [67]. Our EPM experiments were performed 4 days after the last dose of mephedrone administration, therefore we suppose that anxiety observed in our study may be the result of mephedrone withdrawal.

### 3.3. Fear Extinction

Fear extinction is defined as a decline in conditioned fear response following nonreinforced exposure to feared CS. Behavioral evidence indicates that extinction is a form of inhibitory learning. Extinguished fear responses reappear with the passage of time (spontaneous recovery), a shift of context (renewal), and unsignaled presentations of the unconditioned stimulus (reinstatement) [68]. We investigated this possibility through a series of behavioral experiments examining the recoverability of conditioned fear following extinction. Our results indicated that rats exposed to context A exhibit decreasing freezing behavior during four days of extinction procedure. On day 4 of the extinction freezing time of each group of tested animals, the decline was less than 10% of session time. Prior repeated mephedrone administration did not affect the extinction process in the trauma-exposed rats. Mephedrone-treated animals (like non-stressed rats) did not recognize context A as a stressful environment, probably because of mephedrone-induced amnesia. In the subsequent 24 h, the animals were subjected to fear recall in context A with a very low footshock intensity. This stimulus by itself did not induce freezing behavior in non-stressed rats, but reinstated it in stressed animals. Of note, mephedrone-treated stressed rats did not show any freezing behavior. These results suggest that fear memory in saline-treated animals is persistent and, even if it is extinguished, it could be recalled with stimulus even with very low intensity. Furthermore, 24 h later, the animals exposed to context A with no aversive stimulus were found to be still fearful. Repeated mephedrone administration impaired this process.

Researchers show a variety of effects of psychostimulants on fear memory extinctions. However, psychostimulants (e.g., d-amphetamine, methylphenidate, cocaine or modafinil) dose-dependently affect fear memory and learning: they enhance long-term memory at low clinically relevant doses, but impair long-term memory at high, abused doses [69,70,71]. A limitation of our study is that we cannot evaluate the dose-dependent effect of mephedrone administration because we only used one dose of this drug. However, the applied dose did not induce a rewarding effect in non-stressed animals. Therefore, we can suppose that in stressed animals, this dose of mephedrone possesses effects similar to high doses of psychostimulants. Because extinction is a form of new learning dependent on BLA [72,73], one might expect that mephedrone impairs this new learning. Thus, mephedrone cannot be used as a self-medication for PTSD due to its amnestic effects and addiction potential.

### 3.4. Effects of Post-Stress Repeated Mephedrone Exposure on MPP-9, as Well as GluN2A and GluN2B Subunits of NMDA Receptor Expression in Trauma-Susceptible Rats

It has been shown that MMP-9 is involved in fear learning, particularly in contextual fear conditioning [74], which is generally accepted to be disturbed in PTSD [75]. In addition, the MMP-9 inhibitor, doxycycline, was found to significantly reduce experimentally acquired threat memories in a non-clinical sample of human subjects [76]. Although these studies reveal MMP-9 as a promising candidate molecule for PTSD, there are only two studies on MMP-9 expression in PTSD patients. One found an elevation in MMP-9 enzymatic activity in PTSD veterans [77]. In contrast, the other study revealed that serum MMP-9 levels slightly increased in response to stress in the total cohort comprising both PTSD patients and healthy controls (HC), and to be unaltered in PTSD patients, both at baseline and 90 min after exposure to mental stress [78]. On the contrary, a loss in serum MMP-9 after psychosocial stress was reported in HC [79] and in patients with coronary artery disease [80]. Thus, MMP-9 level can depend on the type of stress that is encountered.

In pre-clinical studies, acute psychosocial stress paradigm produces an increment in MMP-9 enzyme levels in the HIPP [81]. In turn, contextual fear conditioning markedly increases MMP-9 transcription, followed by enhanced enzymatic levels in the three major brain structures implicated in fear learning, i.e., the amygdala, HIPP and prefrontal cortex [74]. Our experiments confirm that footshock stress increases MMP-9 expression in saline-treated trauma-exposed rats in the HIPP and BLA. However, mephedrone administration decreased the MMP-9 expression in the HIPP, but not in the BLA. Herein, the decrease of MMP-9 in the HPP can be correlated with memory impairment observed in the mephedrone-treated rats during reconsolidation of fear conditioning [82].

MMP-9 has emerged as a physiological regulator of NMDA receptor-dependent synaptic plasticity and memory [21]. Our results show up-regulation of the GluN2B subunit in the HIPP in trauma-exposed rats that were mephedrone—but not saline-treated before extinction. Thus, such outcome can support reconsolidation of fear memory in saline treated rats after low footshock exposure. A similar effect was observed in trauma-exposed rats in the BLA. Synaptic plasticity mediated by NMDA glutamate receptors is thought to be a primary mechanism underlying the formation of new memories. Published data suggest that GluN2A receptor activation and associated long-term potentiation (LTP) may be involved specifically in the initial formation and/or stabilization of a learned fear response, whereas GluN2B receptor activation and associated long-term depression may facilitate the suppression of Pavlovian fear response during extinction [23]. Up-regulation of the GluN2B subunit can be a response to deficits in glutamate signaling. Thus, an up-regulation of GluN2B subunit in the BLA in mephedrone-treated trauma-exposed rats can be responsible for disruption of the reconsolidation of conditioned fear memory.

In summary, the present study provides evidence that mephedrone abuse may induce retrograde amnesia of PTSD-like fear, similarly to high doses of MDMA. Moreover, trauma-exposed adolescent rats showed stronger mephedrone-induced CPP, indicating that these rats were more sensitive to mephedrone effects. According to our results, repeated mephedrone administration impaired new learning and fear extinction-reconsolidation that could suggest an inability to retrieve warning signals and risk factors. These mephedrone effects involved changes in the MMP-9 and GluN2B subunits of the NMDA receptor in the HIPP and BLA, two brain structures involved in fear learning and memory. However, it is worth mentioning that our study was only done on male adolescent rats, which constitutes one of its major limitations. For our data to gain more translational value, the same experimental procedures need to be applied on female rats as well.

## 4. Materials and Methods

### 4.1. Animals

Adolescent (PND30) male Wistar rats (OMD, Lublin, Poland) weighing 120–170 g at the first day of the study were employed as experimental subjects. All animals (*n* = 28/7 per group) were maintained in cages (55 cm × 33 cm × 20 cm) under standard laboratory conditions (12 h light/dark cycle, room temperature 21 ± 1 °C), and free access to drinking water and standard laboratory chow (Sniff Spezialdiäten GmbH, Soest, Germany). To reduce their stress levels in response to experimental manipulations, rats were handled for 5 min per day for 5 consecutive days prior to initiation of behavioral tests. Experiments were conducted between 8:00 a.m. and 7:00 p.m. All procedures were approved by the Local Ethic Committee (No. 31/2021) and were carried out according to the National Institute of Health Guidelines for the Care and Use of Laboratory Animals of 22 September 2010 (2010/63/EU). The study was conducted in accordance with ARRIVE guidelines.

### 4.2. Drugs

Mephedrone hydrochloride (Tocris Bioscience, Bristol, UK) was dissolved in sterile 0.9% saline (0.9% NaCl, Baxter, Warsaw, Poland). Saline solutions of mephedrone was prepared ex tempore before each administration. Rats were treated intraperitoneally (i.p.), with mephedrone (5 mg/kg) according to previous study [83].

### 4.3. Behavioral Apparatus and Procedures

#### 4.3.1. Fear Conditioning

The PTSD model was induced using a Fear Conditioning apparatus [44]. The animals were placed in a soundproof cage for conditioning (Ugo Basile, Italy) with dimensions of 55 cm × 60 cm × 57 cm. Such a cage is equipped with a light emitting bulb, a loudspeaker and a floor made of metal rods to conduct electricity. The conditioned, neutral stimulus (CS) was the sound signal emitted by the loudspeaker inside the cage, with an intensity of 80 dB and a duration of 28 s. Additionally, the striped walls and the floor provided a context (context A) which, like the sound signal, constituted a CS. The sound signal and the context were associated with the aversive US (the irritation of the animals’ paws with an electric current of 1.5 mA) for the last 2 s after the sound signal. Then, after a short break, the procedure was repeated. Thus, during each training session, the animals were subjected to two sequences of the CS–US. The animal was then left in a soundproof cage for 60 s for memory consolidation, after that, the rat was returned to its home cage. Half of the studied animals (*n* = 14) were treated with electric shock (stressed), while the other half was treated only with CS (non-stressed). The experimental procedures were performed according to previously described methods [84] with modifications. The timeline of the research procedures is presented in Figure 1.

#### 4.3.2. CPP Paradigm

The CPP unbiased paradigm was performed according to the method described earlier [18,83] with minor modifications. The apparatus designed to perform the CPP test consists of 6 cages divided into two compartments, 65 cm × 35 cm × 30 cm in size, differing in the color of the walls. Between the compartments of the cages there is a square opening closed with a guillotine door, which allows the passage of animals between the two sectors of the cage. One of the compartments has solid black, smooth walls, while the other has black and white vertical stripes. The CPP paradigm was performed in three phases, which include: pre-test, conditioning and test.

*Pre-test*. Each test animal was placed randomly in one of the compartments of the apparatus. Rats were allowed to explore both parts for 15 min. This phase of CPP paradigm consisted in determining the time spent by rats in each of the compartments. If the animals showed no primary preference for any of the parts, they were qualified for further phases of the CPP.

*Conditioning*. The qualified animals were conditioned twice daily (morning and afternoon sessions) for five consecutive days with at least a 3 h rest period between sessions. In the morning, all animals were injected with saline and placed into the drug-free compartment for 30 min. In the afternoon, rats received mephedrone (5 mg/kg, i.p.), or equivalent volume of saline and were then placed for 30 min into the drug-paired compartments with the guillotine doors closed.

*CPP test.* The rats were confined individually in the apparatus and left for 15 min, having free access to both compartments. No drugs were administered during this phase. The time spent by the animals in each compartment was measured and recorded.

#### 4.3.3. Elevated Plus Maze (EPM)

This procedure was carried out according to methodology described earlier [84,85,86]. The plus-shaped maze apparatus for rats was made of wood and positioned at a height of 50 cm above the floor in quiet laboratory surroundings. Two opposite arms were open (50 cm × 10 cm) and the other two were enclosed with high walls (50 cm × 10 cm × 40 cm). The level of illumination was approximately 100 lx at floor level of the maze. The experiment was initiated by placing the rat in the center of the plus-maze facing an open arm, after which the number of entries and time spent in each of the two arms were recorded for a period of 5 min. An “arms entry” was recorded when the rat entered the arm with all four paws. The maze was carefully cleaned with 10% *v*/*w* ethanol after each test session to remove the odor cues.

#### 4.3.4. ELISA Assays

Right after the behavioral procedures, the animals were decapitated, and the dissected brain structures were subjected to biochemical experiments to evaluate the influence of PTSD model and mephedrone on MMP-9 and NMDA receptors subunits expression in the BLA and HIPP according to experimental design (Figure 1). Quantitative measurement of MMP-9, GluN2A, and GluN2B in the HIPP and BLA was performed using a rats MMP-9 ELISA Kit (Reddot Biotech, Kelowna, BC, Canada), a rat glutamate [NMDA] receptor subunit epsilon-1 ELISA Kit (E1205Ra; Bioassay Technology Laboratory, Shanghai, China), and a rat glutamate [NMDA] receptor subunit epsilon-2 ELISA Kit (E1204Ra; Bioassay Technology Laboratory, China), respectively, following manufacturers’ protocols. Firstly, frozen rat brain structures were homogenized in ice-cold PBS pH 7.4 containing cocktails of protease and phosphatase inhibitors (Sigma-Aldrich, San Luis, MI, USA) using a homogenizer ball (Bioprep-24, Aosheng, Hangzhou, China) (10 s at rpm). Then, homogenates were centrifuged for 5 min at 5000× *g* and the supernates were immediately removed. Duplicates of each sample and series of standards were transferred to ELISA plates. The absorbance was measured at a wavelength of λ = 450 nm using a Multiskan Spectrum spectrophotometer (Thermo LabSystems, Philadelphia, PA, USA). The concentration of proteins was calculated from a standard curve and expressed as ng/mg of protein. For protein measurement, the bicinchoninic acid assay (BCA) protein assay kit (Serva, Heidelberg, Germany) was used.

### 4.4. Procedures

#### 4.4.1. Stage 1: Impact of Footshock Stress on the Acquisition of Mephedrone-Induced CPP in Adolescent Rats

To evaluate the impact of footshock stress (PTSD model) on the rewarding effect of mephedrone, in the first stage of the experiments, the animals were fear conditioned (day 1, PND30) as described above. Next, following 7 days of the incubation period, on day 8 (PND37), the rats were subjected to CPP procedure. On day 8, the CPP pre-test was performed. According to unbiased procedures, animals that did not show preference to any compartments (half of the animals from the CS and US groups) were assigned to either the mephedrone-conditioned or saline-conditioned group (days 9–14). Ultimately, four research groups were established: (1) Non-stressed/saline; (2) Non-stressed/Mephedrone (5 mg/kg, i.p.); (3) Stressed/Saline; (4) Stressed/Mephedrone (5 mg/kg, i.p.). The mephedrone dose was based on previously published data [83] that show an impact of stress on the rewarding effect of this drug in the CPP paradigm. On day 15 of the experiment the CPP test was conducted (PND44).

#### 4.4.2. Stage 2: Impact of Repeated Mephedrone Injections on Fear Sensitization in Adolescent Rats. Anxiety-like Behavior Based on an EPM Test

On day 16 (PND45), all rats (previously stressed and non-stressed) underwent fear conditioning in a different context B (colorless walls, no signal sound), using free footshocks of 1 s at a lower intensity (0.4 mA). Freezing behavior was recorded (absence of movement except for respiration) and expressed as the percentage of time that the animal spent motionless in the apparatus. This stage of the experiment was performed to evaluate the impact of repeated mephedrone administration on fear memory in the new context B (fear sensitization) of animals previously received footshock. On day 17 (PND46), in the morning, freezing behavior was tested by placing the rats in the context A with the signal sound, but with no electric stimulus. In the afternoon, 6 h after the first test, rats were placed back in the context B (with no tone and shock) and freezing behavior was measured. One day later (day 18, PND47), anxiety-like behavior was assessed in the EPM.

#### 4.4.3. Stage 3: Impact of Repeated Mephedrone Injections on Recall and Retention of Fear Memory in Adolescent Rats

Extinction of fear memory was conducted from day 20 to 23 of the experiment. Each day, the animals were placed in the fear conditioning cage in the context A, but with no exposure to foot shock. The fear memory was considered extinguished when freezing behavior decreased to less than 10% of the time. Next, 24 h later (day 24), the animals were re-exposed to trauma in the context A, but with a very low (0.15 mA) footshock intensity (recall). The final test was conducted in context A with no electric shock at day 25.

### 4.5. Behavioral Scoring and Statistical Analysis

All behavioral procedures were performed and recorded using ANY-maze (Stoelting Co. Wood Dale, IL, USA). The results obtained in the experiment were statistically processed using Graph-Pad Prism 8.3.0 (GraphPad Sofware, San Diego, CA, USA). The statistical analysis of the results was based on two-way and three-way ANOVA. Comparisons between groups were made using the Bonferroni post-hoc test. Differences were considered statistically significant if the determined *p* value was less than 0.05 (*p* < 0.05). The figures show the average values in the studied groups with the standard errors of measurement (±SEM). Results obtained in the CPP paradigm were expressed as scores (the differences between post-test and test time spent in the drug-associated compartment). The results obtained in EPM are presented as the anxiety index, calculated according to the following formula described by Lguensat et al. [85]: 1 − [(time spent in the open arms/total time) + (number of entrances to the open arms/total entries)/2].

## Data Availability

The data presented in this study are available on request from the corresponding author.

## References

[1] Bisson J.I., Cosgrove S., Lewis C., Robert N.P. (2015). Post-traumatic stress disorder. BMJ.

[2] Shalev A.Y. (2009). Posttraumatic stress disorder and stress-related disorders. Psychiatr. Clin. N. Am..

[3] Franke L.K., Rattel J.A., Miedl S.F., Danböck S.K., Bürkner P.C., Wilhelm F.H. (2021). Intrusive memories as conditioned responses to trauma cues: An empirically supported concept?. Behav. Res. Ther..

[4] Wessa M., Flor H. (2007). Failure of extinction of fear responses in posttraumatic stress disorder: Evidence from second-order conditioning. Am. J. Psychiatry.

[5] Seidemann R., Duek O., Jia R., Levy I., Harpaz-Rotem I. (2021). The Reward System and Post-Traumatic Stress Disorder: Does Trauma Affect the Way We Interact with Positive Stimuli?. Chronic Stress.

[6] Guina J., Rossetter S.R., De Rhodes B.J., Nahhas R.W., Welton R.S. (2015). Benzodiazepines for PTSD: A systematic review and meta-analysis. J. Psychiatr. Pract..

[7] Tipps M.E., Raybuck J.D., Buck K.J., Lattal K.M. (2014). Delay and trace fear conditioning in C57BL/6 and DBA/2 mice: Issues of measurement and performance. Learn. Mem..

[8] Basedow L.A., Kuitunen-Paul S., Wiedmann M.F., Roessner V., Golub Y. (2021). Self-reported PTSD is associated with increased use of MDMA in adolescents with substance use disorders. Eur. J. Psychotraumatol..

[9] Mitchell J.M., Bogenschutz M., Lilienstein A., Harrison C., Kleiman S., Parker-Guilbert K., Ot’alora G.M., Garas W., Paleos C., Gorman I. (2021). MDMA-assisted therapy for severe PTSD: A randomized, double-blind, placebo-controlled phase 3 study. Nat. Med..

[10] Brady K.T., Sinha R. (2005). Co-occurring mental and substance use disorders: The neurobiological effects of chronic stress. Am. J. Psychiatry.

[11] Cleck J.N., Blendy J.A. (2008). Making a bad thing worse: Adverse effects of stress on drug addiction. J. Clin. Investig..

[12] Koob G.F., Le Moal M. (2008). Addiction and the brain antireward system. Annu. Rev. Psychol..

[13] Brandt S.D., Sumnall H.R., Measham F., Cole J. (2010). Analyses of second-generation ‘legal highs’ in the UK: Initial findings. Drug Test. Anal..

[14] Hockenhull J., Murphy K.G., Paterson S. (2016). Mephedrone use is increasing in London. Lancet.

[15] Angoa-Pérez M., Kane M.J., Francescutti D.M., Sykes K.E., Shah M.M., Mohammed A.M., Thomas D.M., Kuhn D.M. (2012). Mephedrone, an abused psychoactive component of ‘bath salts’ and methamphetamine congener, does not cause neurotoxicity to dopamine nerve endings of the striatum. J. Neurochem..

[16] Papaseit E., Moltó J., Muga R., Torrens M., de la Torre R., Farré M. (2017). Clinical pharmacology of the synthetic cathinone mephedrone. Curr. Top. Behav. Neurosci..

[17] Grochecki P., Smaga I., Lopatynska-Mazurek M., Gibula-Tarlowska E., Kedzierska E., Listos J., Talarek S., Marszalek-Grabska M., Hubalewska-Mazgaj M., Korga-Plewko A. (2021). Effects of mephedrone and amphetamine exposure during adolescence on spatial memory in adulthood: Behavioral and neurochemical analysis. Int. J. Mol. Sci..

[18] Grochecki P., Smaga I., Marszalek-Grabska M., Lopatynska-Mazurek M., Slowik T., Gibula-Tarlowska E., Kedzierska E., Listos J., Filip M., Kotlinska J.H. (2022). Alteration of ethanol reward by prior mephedrone exposure: The role of age and matrix metalloproteinase-9 (MMP-9). Int. J. Mol. Sci..

[19] Papaseit E., Pérez-Mañá C., de Sousa Fernandes Perna E.B., Olesti E., Mateus J., Kuypers K.P.C., Theunissen E.L., Fonseca F., Torrens M., Ramaekers J.G. (2020). Mephedrone and alcohol interactions in humans. Front. Pharmacol..

[20] Martinelli S., Anderzhanova E.A., Bajaj T., Wiechmann S., Dethloff F., Weckmann K., Heinz D.E., Ebert T., Hartmann J., Geiger T.M. (2021). Stress-primed secretory autophagy promotes extracellular BDNF maturation by enhancing MMP9 secretion. Nat. Commun..

[21] Michaluk P., Mikasova L., Groc L., Frischknecht R., Choquet D., Kaczmarek L. (2009). Matrix metalloproteinase-9 controls NMDA receptor surface diffusion through integrin beta1 signaling. J. Neurosci..

[22] Chambers R.A., Bremner J.D., Moghaddam B., Southwick S.M., Charney D.S., Krystal J.H. (1999). Glutamate and post-traumatic stress disorder: Toward a psychobiology of dissociation. Semin. Clin. Neuropsychiatry.

[23] Dalton G.L., Wu D.C., Wang Y.T., Floresco S.B., Phillips A.G. (2012). NMDA GluN2A and GluN2B receptors play separate roles in the induction of LTP and LTD in the amygdala and in the acquisition and extinction of conditioned fear. Neuropharmacology.

[24] Bienvenu T.C.M., Dejean C., Jercog D., Aouizerate B., Lemoine M., Herry C. (2021). The advent of fear conditioning as an animal model of post-traumatic stress disorder: Learning from the past to shape the future of PTSD research. Neuron.

[25] Johnson L.R., McGuire J., Lazarus R., Palmer A.A. (2012). Pavlovian fear memory circuits and phenotype models of PTSD. Neuropharmacology.

[26] Ross D.A., Arbuckle M.R., Travis M.J., Dwyer J.B., van Schalkwyk G.I., Ressler K.J. (2017). An integrated neuroscience perspective on formulation and treatment planning for posttraumatic stress disorder: An educational review. JAMA Psychiatry.

[27] Kim J.J., Fanselow M.S. (1992). Modality-specific retrograde amnesia of fear. Science.

[28] Phillips R.G., LeDoux J.E. (1992). Differential contribution of amygdala and hippocampus to cued and contextual fear conditioning. Behav. Neurosci..

[29] Rudy J.W. (1993). Contextual conditioning and auditory cue conditioning dissociate during development. Behav. Neurosci..

[30] Logue S.F., Paylor R., Wehner J.M. (1997). Hippocampal lesions cause learning deficits in inbred mice in the Morris water maze and conditioned-fear task. Behav. Neurosci..

[31] Caprioli D., Celentano M., Paolone G., Badiani A. (2007). Modeling the role of environment in addiction. Prog. Neuro-Psychopharmacol. Biol. Psychiatry.

[32] Le Moal M. (2009). Drug abuse: Vulnerability and transition to addiction. Pharmacopsychiatry.

[33] Miczek K.A., Yap J.J., Covington H.E. (2008). Social stress, therapeutics and drug abuse: Preclinical models of escalated and depressed intake. Pharmacol. Ther..

[34] Sinha R. (2011). New findings on biological factors predicting addiction relapse vulnerability. Curr. Psychiatry Rep..

[35] Burke A.R., Miczek K.A. (2014). Stress in adolescence and drugs of abuse in rodent models: Role of dopamine, CRF, and HPA axis. Psychopharmacology.

[36] Koob G.F., Volkow N.D. (2010). Neurocircuitry of addiction. Neuropsychopharmacology.

[37] Rodríguez-Arias M., Valverde O., Daza-Losada M., Blanco-Gandía M.C., Aguilar M.A., Miñarro J. (2013). Assessment of the abuse potential of MDMA in the conditioned place preference paradigm: Role of CB1 receptors. Prog. Neuro-Psychopharmacol. Biol. Psychiatry.

[38] Sinha R. (2008). Chronic stress, drug use, and vulnerability to addiction. Ann. N. Y. Acad. Sci..

[39] Bahi A., Dreyer J.L. (2014). Chronic psychosocial stress causes delayed extinction and exacerbates reinstatement of ethanol-induced conditioned place preference in mice. Psychopharmacology.

[40] Filarowska-Jurko J., Komsta L., Smaga I., Surowka P., Marszalek-Grabska M., Grochecki P., Nizio D., Filip M., Kotlinska J.H. (2022). Maternal separation alters ethanol drinking and reversal learning processes in adolescent rats: The impact of sex and glycine transporter type 1 (GlyT1) inhibitor. Int. J. Mol. Sci..

[41] Cannady R., Nguyen T., Padula A.E., Rinker J.A., Lopez M.F., Becker H.C., Woodward J.J., Mulholland P.J. (2021). Interaction of chronic intermittent ethanol and repeated stress on structural and functional plasticity in the mouse medial prefrontal cortex. Neuropharmacology.

[42] Barchiesi R., Chanthongdee K., Domi E., Gobbo F., Coppola A., Asratian A., Toivainen S., Holm L., Augier G., Xu L. (2021). Stress-induced escalation of alcohol self-administration, anxiety-like behavior, and elevated amygdala Avp expression in a susceptible subpopulation of rats. Addict. Biol..

[43] Enman N.M., Arthur K., Ward S.J., Perrine S.A., Unterwald E.M. (2015). Anhedonia, reduced cocaine reward, and dopamine dysfunction in a rat model of posttraumatic stress disorder. Biol. Psychiatry.

[44] Lguensat A., Montanari C., Vielle C., Bennis M., Ba-M’hamed S., Baunez C., Garcia R. (2021). Repeated cocaine exposure prior to fear conditioning induces persistency of PTSD-like symptoms and enhancement of hippocampal and amygdala cell density in male rats. Brain Struct. Funct..

[45] Eagle A.L., Perrine S.A. (2013). Methamphetamine-induced behavioral sensitization in a rodent model of posttraumatic stress disorder. Drug Alcohol Depend..

[46] Spanagel R., Noori H.R., Heilig M. (2014). Stress and alcohol interactions: Animal studies and clinical significance. Trends Neurosci..

[47] Weera M.M., Gilpin N.W. (2019). Biobehavioral interactions between stress and alcohol. Alcohol. Res..

[48] Burke A.R., Watt M.J., Forster G.L. (2011). Adolescent social defeat increases adult amphetamine conditioned place preference and alters D2 dopamine receptor expression. Neuroscience.

[49] Hymel K.A., Eans S.O., LSitchenko K., Gomes S.M., Lukowsky A.L., Medina J.M., Sypek E.I., Carey A.N., McLaughlin J.P. (2014). Stress-induced increases in depression-like and cocaine place-conditioned behaviors are reversed by disruption of memories during reconsolidation. Behav. Pharmacol..

[50] McLaughlin J.P., Li S., Valdez J., Chavkin T.A., Chavkin C. (2006). Social defeat stress-induced behavioral responses are mediated by the endogenous kappa opioid system. Neuropsychopharmacology.

[51] Marquardt A.R., Ortiz-Lemos L., Lucion A.B., Barros H.M. (2004). Influence of handling or aversive stimulation during rats’ neonatal or adolescence periods on oral cocaine self-administration and cocaine withdrawal. Behav. Pharmacol..

[52] Piazza P.V., Deminiere J.M., le Moal M., Simon H. (1990). Stress- and pharmacologically-induced behavioral sensitization increases vulnerability to acquisition of amphetamine self-administration. Brain Res..

[53] García-Pardo M.P., Blanco-Gandía M.C., Valiente-Lluch M., Rodríguez-Arias M., Miñarro J., Aguilar M.A. (2015). Long-term effects of repeated social stress on the conditioned place preference induced by MDMA in mice. Prog. Neuro-Psychopharmacol. Biol. Psychiatry.

[54] Siegmund A., Wotjak C.T. (2007). A mouse model of posttraumatic stress disorder that distinguishes between conditioned and sensitised fear. J. Psychiatr. Res..

[55] Cohen H., Zohar J., Matar M.A., Zeev K., Loewenthal U., Richter-Levin G. (2004). Setting apart the affected: The use of behavioral criteria in animal models of post traumatic stress disorder. Neuropsychopharmacology.

[56] Bentefour Y., Bennis M., Garcia R., M’hamed S.B. (2015). Effects of paroxetine on PTSD-like symptoms in mice. Psychopharmacology.

[57] Bentefour Y., Bennis M., Garcia R., Ba-M’hamed S. (2018). High-frequency stimulation of the infralimbic cortex, following behavioral suppression of PTSD-like symptoms, prevents symptom relapse in mice. Brain Stimul..

[58] Lewis M.C., Gould T.J. (2003). Nicotine and ethanol enhancements of acoustic startle reflex are mediated in part by dopamine in C57BL/6J mice. Pharmacol. Biochem. Behav..

[59] Perusini J.N., Meyer E.M., Long V.A., Rau V., Nocera N., Avershal J., Maksymetz J., Spigelman I., Fanselow M.S. (2016). Induction and expression of fear sensitization caused by acute traumatic stress. Neuropsychopharmacology.

[60] Olatunji B.O., Fan Q., Wolitzky-Taylor K. (2018). Anxiety sensitivity and post-traumatic stress reactions: Effects of time-varying intrusive thoughts and associated distress. J. Behav. Ther. Exp. Psychiatry.

[61] Marshall G.N., Miles J.N., Stewart S.H. (2010). Anxiety sensitivity and PTSD symptom severity are reciprocally related: Evidence from a longitudinal study of physical trauma survivors. J. Abnorm. Psychol..

[62] Ross E.A., Reisfield G.M., Watson M.C., Chronister C.W., Goldberger B.A. (2012). Psychoactive “bath salts” intoxication with methylenedioxypyrovalerone. Am. J. Med..

[63] Valente M.J., Guedes de Pinho P., de Lourdes Bastos M., Carvalho F., Carvalho M. (2014). Khat and synthetic cathinones: A review. Arch. Toxicol..

[64] Perrine S.A., Sheikh I.S., Nwaneshiudu C.A., Schroeder J.A., Unterwald E.M. (2008). Withdrawal from chronic administration of cocaine decreases delta opioid receptor signaling and increases anxiety- and depression-like behaviors in the rat. Neuropharmacology.

[65] Kitanaka N., Kitanaka J., Watabe K., Takemura M. (2010). Low-dose pretreatment with clorgyline decreases the levels of 3-methoxy-4-hydroxyphenylglycol in the striatum and nucleus accumbens and attenuates methamphetamine-induced conditioned place preference in rats. Neuroscience.

[66] El Hage C., Rappeneau V., Etievant A., Morel A.L., Scarna H., Zimmer L., Bérod A. (2012). Enhanced anxiety observed in cocaine withdrawn rats is associated with altered reactivity of the dorsomedial prefrontal cortex. PLoS ONE.

[67] Xu P., Qiu Y., Zhang Y., Βai Y., Xu P., Liu Y., Kim J.H., Shen H.W. (2016). The effects of 4-methylethcathinone on conditioned place preference, locomotor sensitization, and anxiety-like behavior: A comparison with methamphetamine. Int. J. Neuropsychopharmacol..

[68] Myers K.M., Davis M. (2007). Mechanisms of fear extinction. Mol. Psychiatry.

[69] Wood S.C., Fay J., Sage J.R., Anagnostaras S.G. (2007). Cocaine and Pavlovian fear conditioning: Dose-effect analysis. Behav. Brain Res..

[70] Shuman T., Wood S.C., Anagnostaras S.G. (2009). Modafinil and memory: Effects of modafinil on Morris water maze learning and Pavlovian fear conditioning. Behav. Neurosci..

[71] Carmack S.A., Howell K.K., Rasaei K., Reas E.T., Anagnostaras S.G. (2014). Animal model of methylphenidate’s long-term memory-enhancing effects. Learn Mem..

[72] Maren S., Quirk G.J. (2004). Neuronal signalling of fear memory. Nat. Rev. Neurosci..

[73] Pape H.C., Pare D. (2010). Plastic synaptic networks of the amygdala for the acquisition, expression, and extinction of conditioned fear. Physiol. Rev..

[74] Ganguly K., Rejmak E., Mikosz M., Nikolaev E., Knapska E., Kaczmarek L. (2013). Matrix metalloproteinase (MMP) 9 transcription in mouse brain induced by fear learning. J. Biol. Chem..

[75] VanElzakker M.B., Dahlgren M.K., Davis F.C., Dubois S., Shin L.M. (2014). From Pavlov to PTSD: The extinction of conditioned fear in rodents, humans, and anxiety disorders. Neurobiol. Learn. Mem..

[76] Bach D.R., Tzovara A., Vunder J. (2018). Blocking human fear memory with the matrix metalloproteinase inhibitor doxycycline. Mol. Psychiatry.

[77] Brahmajothi M.V., Abou-Donia M.B. (2020). PTSD susceptibility and challenges: Pathophysiological consequences of behavioral symptoms. Mil. Med..

[78] Lima B.B., Hammadah M., Wilmot K., Pearce B.D., Shah A., Levantsevych O., Kaseer B., Obideen M., Gafeer M.M., Kim J.H. (2019). Posttraumatic stress disorder is associated with enhanced interleukin-6 response to mental stress in subjects with a recent myocardial infarction. Brain Behav. Immun..

[79] Szymanowski A., Nijm J., Kristenson M., Jonasson L. (2011). Elevated levels of circulating matrix metalloproteinase-9 are associated with a dysregulated cortisol rhythm--A case-control study of coronary artery disease. Psychoneuroendocrinology.

[80] Lundberg A.K., Jönsson S., Stenmark J., Kristenson M., Jonasson L. (2016). Stress-induced release of matrix metalloproteinase-9 in patients with coronary artery disease: The possible influence of cortisol. Psychoneuroendocrinology.

[81] Aguayo F.I., Pacheco A.A., García-Rojo G.J., Pizarro-Bauerle J.A., Doberti A.V., Tejos M., García-Pérez M.A., Rojas P.S., Fiedler J.L. (2018). Matrix metalloproteinase 9 displays a particular time response to acute stress: Variation in its levels and activity distribution in rat hippocampus. ACS Chem. Neurosci..

[82] Brown T.E., Wilson A.R., Cocking D.L., Sorg B.A. (2009). Inhibition of matrix metalloproteinase activity disrupts reconsolidation but not consolidation of a fear memory. Neurobiol. Learn. Mem..

[83] Wronikowska O., Zykubek M., Kurach Ł., Michalak A., Boguszewska-Czubara A., Budzyńska B. (2021). Vulnerability factors for mephedrone-induced conditioned place preference in rats-the impact of sex differences, social-conditioning and stress. Psychopharmacology.

[84] Lguensat A., Boudjafad Z., Giorla E., Bennis M., Baunez C., Garcia R., Ba-M’hamed S. (2021). Repeated ethanol exposure following avoidance conditioning impairs avoidance extinction and modifies conditioning-associated prefrontal dendritic changes in a mouse model of post-traumatic stress disorder. Eur. J. Neurosci..

[85] Koltunowska D., Gibula-Bruzda E., Kotlinska J.H. (2013). The influence of ionotropic and metabotropic glutamate receptor ligands on anxiety-like effect of amphetamine withdrawal in rats. Prog. Neuro-Psychopharmacol. Biol. Psychiatry.

[86] Gibula-Bruzda E., Marszalek-Grabska M., Witkowska E., Izdebski J., Kotlinska J.H. (2015). Enkephalin analog, cyclo[N(ε),N(β)-carbonyl-D-Lys(2),Dap(5)] enkephalinamide (cUENK6), inhibits the ethanol withdrawal-induced anxiety-like behavior in rats. Alcohol.

